# Association of attrition with mortality: Findings from 11 waves over three decades of the Whitehall II study

**DOI:** 10.1136/jech-2019-213175

**Published:** 2020-06-25

**Authors:** Mifuyu Akasaki, Mika Kivimäki, Andrew Steptoe, Owen Nicholas, Martin J Shipley

**Affiliations:** 1. Department of Behavioural Science and Health, University College London, 1-19 Torrington Place, London WC1E 6BT United Kingdom; 2. Department of Epidemiology and Public Health, University College London, 1-19 Torrington Place, London WC1E 6BT United Kingdom; 3. Department of Statistical Science, University College London, 1-19 Torrington Place, London WC1E 6BT United Kingdom

**Keywords:** Attrition, withdrawal, non-response, longitudinal study, selection bias, collider bias

## Abstract

**Background:**

Attrition, the loss of participants as a study progresses, is a considerable challenge in longitudinal studies. This study examined whether two forms of attrition, *“withdrawal”* (formal discontinued participation) and *“non-response”* (non-response among participants continuing in the study) have different associations with mortality, and whether these associations differed across time in a multi-wave longitudinal study.

**Methods:**

Participants were 10 012 civil servants who participated at the baseline of the Whitehall II cohort study with 11 data waves over average follow-up of 28 years. We performed competing-risks analyses to estimate sub-distribution hazard ratios and 95% confidence intervals, and likelihood ratio tests to examine whether hazards differed between the two forms of attrition. We then applied linear regression to examine any trend of hazards against time.

**Results:**

Attrition rate at data collections ranged between 13% and 34%. There were 495 deaths recorded from cardiovascular disease and 1367 deaths from other causes. Study participants lost due to attrition had 1.55 (95% confidence interval 1.26 to 1.89) and 1.56 (1.39 to 1.76) times higher hazard of cardiovascular and non-cardiovascular mortality than responders respectively. Hazards for withdrawal and non-response did not differ for either cardiovascular (p-value = 0.28) or non-cardiovascular mortality (p-value = 0.38). There was no linear trend in hazards over the 11 waves (cardiovascular mortality p-value = 0.11, non-cardiovascular mortality p-value = 0.61).

**Conclusion:**

Attrition can be a problem in longitudinal studies resulting in selection bias. Researchers should examine the possibility of selection bias and consider applying statistical approaches that minimise this bias.

## Introduction

Many long-term cohort studies are affected by gradual attrition due to withdrawal and non-response (1). One challenge is to ensure that inferences drawn are applicable to the members of the study population; internal validity (2, 3). If some study participants do not respond and they have systematically different characteristics from those who do, then estimated effects among the responders may not pertain to the original study population (4, 5). In this situation, estimation may be biased, thus undermining external validity or generalisability (2, 3). In addition, ensuring internal and external validity are important challenges for researchers as response rates in studies have generally declined over the past four decades, possibly because of increased burden on participants (e.g. increase in the number of studies, more extensive and time-consuming questionnaires, biological sampling, the requirements of participants’ consent) (6, 7).

Studies have investigated characteristics of non-responders to understand predictors of non-response, and potential for bias in results. For instance, those who drop out from studies are more likely to be men (8–10), be young or old people (11, 12), be single (8, 13), be in a lower employment grade (14, 15), have adverse smoking or alcohol drinking habits (16, 17), have greater cognitive impairment (10, 18), and have worse health (14, 19). Analysis of the Whitehall II study, a large multi-wave cohort study, has shown differences in characteristics of participants when distinguishing between response, non-response, or withdrawal - the three categories of “response status” - of a participant (8). Withdrawers from the study were more likely to have adverse mental health, while non-responders were less likely to have long-standing illnesses (8). Not only is it important for this study, and others, to recognise and compensate for those at higher risk being under-represented in participants (20), but also it is important to address whether there are clear differences in risk by category of attrition, and if so why.

Population-based studies linked with electronic health records suggest that attrition is associated with an approximate doubling of the risk of mortality (16, 21, 22). To date, most relevant studies have employed response status at a single time point (i.e. baseline), with no distinction between withdrawals and non-responders, or have used patterns of response status over time (21, 23). It is unclear whether the association of attrition with higher mortality applies only to non-responders at baseline, or whether the association persists and applies to all waves. If there were a trend in the risk of mortality in responders compared to the risk in those lost to attrition even after adjustment for measured factors such as age, it would be a sign that differences in unmeasured risk factors between responders and those lost to attrition change wave to wave; hence a sign that sources of bias change wave to wave (24). Some studies have examined trends in mortality over time by baseline response status, but failed to consider response status at follow-up (22, 25). Furthermore, it is unknown whether attrition is associated with increased mortality in CVD, a major cause of death.

Accordingly, this study aims to (i) examine the extent to which response status at each wave is associated with cardiovascular and non-cardiovascular mortality up to the following wave; (ii) investigate whether the hazard of mortality differs between two forms of attrition: withdrawal, and non-response; and (iii) assess whether there is a trend across waves in the association between attrition and mortality.

## Methods

### Study population

**Figure 1.**
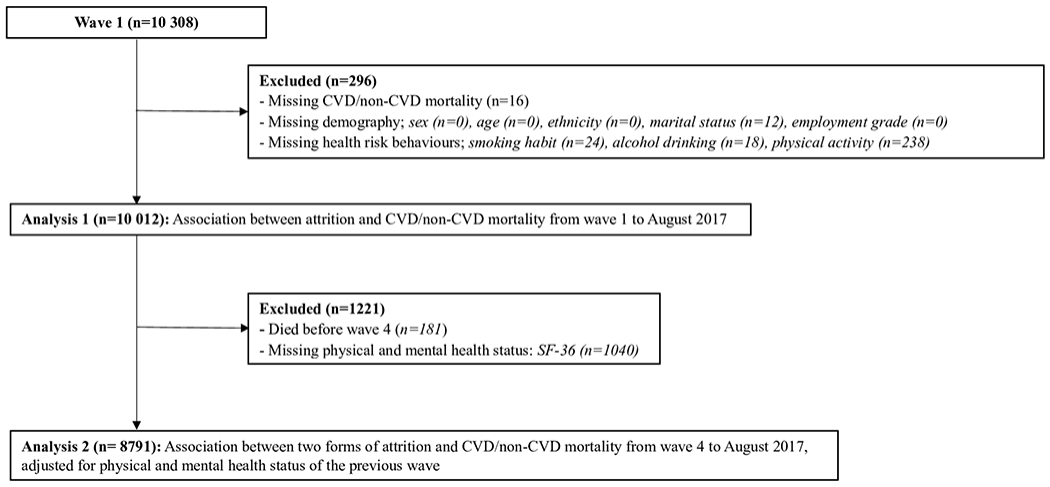
Flow chart of participants’ recruitment

The Whitehall II study was established in 1985 to determine the factors which contribute to social inequalities in health. There were 10 308 participants (men 6895; women 3413, aged 33–55) at entry to the study (wave 1) who were non-industrial civil servants from 20 Civil Service Departments in London. The study has had twelve waves of data collection up to 2016. The response rate in each wave has remained over 65% across all waves separated by three years on average. We included 10 012 participants who responded at baseline and who have no missing values in covariates and mortality (Figure 1).

### Variables

#### Response status

The Whitehall II study has conducted both self-administered questionnaires and medical examinations at odd-numbered waves, and self-administered questionnaires only at even-numbered waves, In our analysis, for each study participant at each wave, “response” is when the participant either completes the self-administered questionnaire or attends the medical examinations at a wave. “Withdrawal” is when the participant officially informs the study research team that they wish to permanently leave the study, and “non-response” is when the participant (who has not formally withdrawn from the study) does not respond at a certain wave. Participants who have withdrawn from the study are not contacted again at future waves whereas non-responders are re-contacted and could participate at later waves. Non-response is not due to mortality. We term either withdrawal or non-response as “attrition”, and “response status” as comprising response and attrition. Prior to wave 4 it is not possible to distinguish withdrawal from non-response due to the way how the data were collected. We therefore conducted two analyses. In analysis 1, we used all waves from wave 1 in terms of attrition (i.e. withdrawal or non-response combined) and in Analysis 2 we analysed data from wave 4 onwards, using all three categories of response status (i.e. withdrawal, non-response, response). Reasons for withdrawal and non-response were not available.

#### Mortality

Cardiovascular disease (CVD) and non-CVD mortality were tracked by the National Health Services (NHS) central registry. CVD mortality includes coronary heart disease, angina, myocardial infarction and stroke. Mortality was tracked from wave 1 to August 2017 in 10 292 participants (99.8%), with mean follow-up of 28.7 years (standard deviation: 5.1 years). We identified CVD mortality based on International Classification of Disease (ICD)-9 (codes 390-459) and 10 (codes I00 - I99). Non-CVD mortality includes cancer (ICD-9: 140-239; 10: C00-C97), respiratory mortality (ICD-9: 460-519; 10: J00-J99) and any other cause not classified as CVD mortality.

#### Covariates

We adjusted for factors related to sociodemographic characteristics, health risk behaviours, and general health status to examine whether these could explain the associations between response status and mortality. Covariates were available only when response status was “response” and therefore present for all participants only at wave 1. We measured covariates using standard questionnaire measures.

#### Sociodemographic characteristics

Participants’ sex, age in years, ethnicity (white vs. non-white), marital status (married/cohabiting, single, divorced/widowed) and employment grade are all associated with health (26) and were taken from the first wave of the study. Information on sex, age, and employment grade at wave 1 was known for all participants. Missing values in ethnicity and marital status were replaced, where known, with responses from the wave 5 and wave 2 questionnaire respectively. Employment grade was categorised as “administrative” (high grade), “professional/executive” (intermediate grade), and “clerical/support” (low grade).

#### Health risk behaviours

Health behaviours were taken from participants’ questionnaire responses at wave 1 of the study. Smoking habit (never-smoker, ex-smoker, and current-smoker), alcohol drinking (<14 units per week and ≥14 and over units per week), and leisure-time physical activity (high, intermediate, low) were included. Physical activity was assessed based on answers to questions about the frequency and duration of participation in moderately energetic (e.g. dancing, cycling, leisurely swimming), and vigorous physical activity (e.g. running, hard swimming, playing squash). Missing values were replaced with those from the waves 2 and 3. The cut-off points for alcohol consumption and physical activity were determined in line with the NHS guideline (27).

#### General health status

The 36-item Short Form Health Survey (SF-36) physical – Physical Component Score (PCS) - and mental – Mental Component Score (MCS) - scores were included. PCS is derived from; general health perceptions (5 items), physical functioning (10 items), role limitations due to physical functioning (4 items), bodily pain (2 items). MCS is derived from; vitality (4 items), general mental health (5 items), role limitations due to emotional problems (3 items), and social functioning (2 items). Higher scores represent better health. PCS and MCS are not available prior to wave 3 and were therefore omitted from Analysis 1. Analysis 2 treated PCS and MCS from the previous wave as covariates. Missing PCS and MCS values were replaced using the last known measurement carried forward. We categorised PCS and MCS using wave- and sex-specific quartiles.

### Statistical methods

We calculated participants’ response rate across all waves of the study as the number of waves responded divided by the number of waves that they could have responded to while still alive (28). Mean response rates and 95% confidence intervals (CIs) by levels of each covariate were calculated.

We used competing-risks analysis to assess the association of subsequent mortality with the time scale being study wave, with attrition status (analysis 1) or response status (analysis 2) at each wave as the exposure. The sub-distribution hazard ratios (SHRs) and 95% CIs of CVD mortality were estimated using non-CVD mortality as a competing risk. Similarly, those of non-CVD mortality were estimated with CVD mortality as a competing risk. We included interaction terms between attrition/response status and sex, age, and employment grade, to assess whether these factors modified associations between attrition/response status and mortality. We also investigated whether SHRs showed evidence of trend across waves by regressing point estimates of SHRs against wave. We conducted two analyses as follows (Figure 1).

#### Analysis 1:

We analysed 10 012 participants, initially for the association of attrition status with CVD and non-CVD mortality from wave 1 up to August 2017, adjusted for sex and age, and finally additionally adjusting for marital status, ethnicity, employment grade, smoking, alcohol drinking, and physical activity.

#### Analysis 2:

In 8791 participants we analysed the association of response status with CVD and non-CVD mortality, from wave 4 up to August 2017, adjusting as in analysis 1 with the addition of PCS and MCS from the previous wave as time-varying variables. In this analysis, we included participants who had responses in both PCS and MCS from at least one wave between wave 3 and wave 11. Likelihood ratio tests were used to examine whether the estimated risks of mortality differ across the two forms of attrition by comparing models of attrition status with models of response status.

We conducted sensitivity analyses by repeating analysis 1 using person-years, rather than wave, as the time scale in the same models as used in the main analysis.

We used the Stata SE version 15.1 for all analyses.

## Results

The total number of participants recruited into the Whitehall II study at wave 1 was 10 308, and their response status at each wave is given in Table 1. The attrition rate was between one fifth and one third of eligible study population (those who had not died) at each wave except at waves 3 and 4 when efforts were made to raise participation. The proportion of deaths attributable to CVD rose, then fell, as research participants aged. In analysis 1, we included 10 012 participants, who had no missing values in covariates, CVD, and non-CVD mortality (men; 67.4%). Table 2 shows the participants’ response rates (the proportion of waves attended) according to the characteristics of study population. Response rates were higher in men (81.9%) than women (74.0%), and showed a trend across employment grade, being highest in the highest grade (86.1%) and lowest in the lowest grade (66.2%).

Table 3 shows the association between attrition status and CVD and non-CVD mortality. There were 495 deaths recorded from CVD and 1367 deaths from non-CVD. Compared to responders, participants with attrition had 1.55 (95% CI 1.26 to 1.89) times the hazard of CVD mortality after adjustment for sex, age, ethnicity, marital status, employment grade, smoking habit, alcohol drinking, and physical activity. For non-CVD mortality, the hazard ratio was 1.56 (1.39 to 1.76). The association between attrition and mortality was not modified by sex, age, or employment grade. Table S1 in the online supplementary file shows the SHRs and 95% CIs for the association between attrition and CVD and non-CVD mortality from each wave to the following wave, on average a period of three years. There was no evidence of trend in point estimates of SHRs across the waves for either CVD mortality (p-value = 0.11) or for non-CVD mortality (p-value = 0.61). Sensitivity analyses using person-years, rather than wave, showed the same pattern of results, but with all the SHRs slightly reduced (Table S2, online supplementary material).

**Figure 2.**
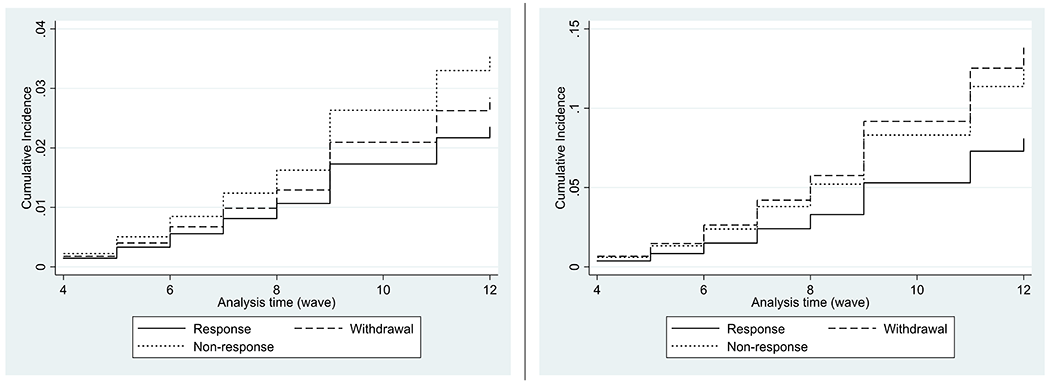
Cumulative incidence function of CVD and Non-CVD mortality by response status (left; CVD mortality, right; non-CVD mortality)

**Figure 3.**
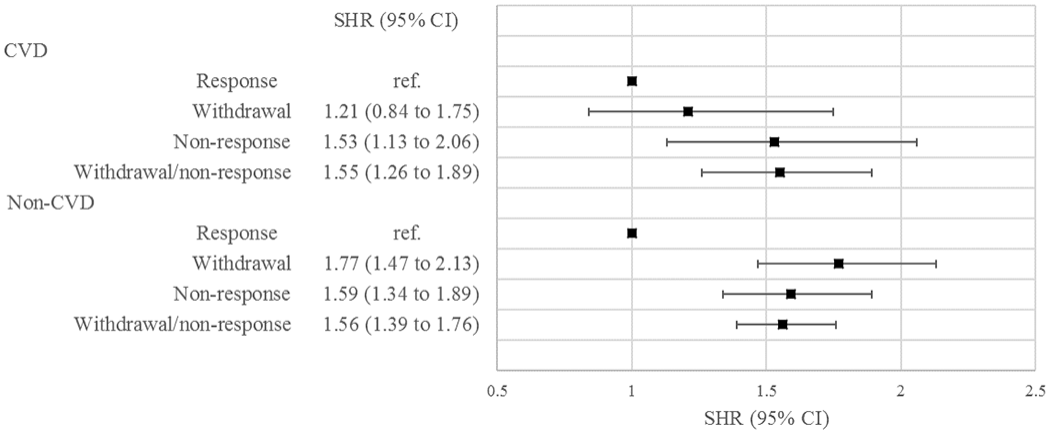
Sub-distribution Hazard Ratios (SHRs)^a^ and 95% Confidence Intervals (CIs) of CVD and Non-CVD mortality by response status ^a^ SHRs of *withdrawal/non-response* are based on 10 012 participants (analysis 1), adjusted for sex, age, ethnicity, marital status, employment grade, smoking habit, alcohol drinking, and physical activity. SHRs of *withdrawal* and *non-response* are based on 8791 participants (analysis 2), adjusting as in analysis 1 with the addition of PCS and MCS.

From wave 4 onwards, attrition could be partitioned into non-responders and those who had completely withdrawn from the study. Among 8791 participants in analysis 2, there were 353 deaths recorded from CVD and 1056 deaths from other causes. Figure 2 shows the cumulative incidence function (CIF) for CVD and non-CVD mortality from wave 4 for each response status. For CVD mortality, the curves of CIF between non-response and withdrawal diverged, whilst for non-CVD mortality those between non-response and withdrawal were almost parallel. The association of response status with mortality is shown graphically in the figure 3, and further details of the results are given in Tables S3, S4, and S5 in the online supplementary material. Likelihood ratio tests showed no evidence that the differentiation of two types of attrition improved the models for either CVD (p-value = 0.28) or non-CVD mortality (p-value = 0.38).

## Discussion

The principal findings are that, compared to responders, attrition after baseline is associated with approximately 1.5 times higher hazard of mortality for both CVD and non-CVD mortality after adjustment for covariates. There is no difference in the hazard of either CVD or non-CVD mortality between withdrawal and non-response. In addition, the association of attrition with mortality does not vary across waves.

Our findings show a slightly weaker association than previous studies, which have reported a doubling of the hazard of mortality in those with attrition compared to responders (16, 21, 22). This may be because previous studies categorised response status retrospectively from deaths as an end point, while we used prospectively measured response status; or because the majority used response status at baseline only, not during follow-up. It may be explained by the previous findings that non-responders at baseline had a remarkably higher hazard of mortality than participants in longitudinal studies (16, 21, 22, 25). We found no differences in the hazard between withdrawal and non-response, our null hypothesis. A possible explanation is that, among those lost due to attrition, the two distributions of reasons for attrition, between withdrawals and non-responders, do not differ across the waves. The associations of response status with CVD mortality were attenuated with adjustment for sociodemographic factors and health risk behaviours, consistent with the previous studies (8–18, 29–31). Morbidity is also one of the potential predictors of attrition. Some (14, 19, 32), but not all (8) of the literature has documented that those who have illness are more likely to be lost to follow-up. To examine this association, we included physical and mental health status using SF-36 from the previous wave in the model. However, it did not attenuate the association, possibly because it may depend on the severity of illness, whether illness is acute or chronic, or the existence of psychological illness, rather than general health status.

The association between response status and subsequent mortality is not causal; however, as our study shows, response status may predict mortality in later waves. This implies that internal and external validity of studies may be affected in certain circumstances (4, 33, 34). For example, selection can lead to collider bias (a bias occurring when two variables independently affect a third variable, and that third variable is conditioned upon), which can bias estimations (4). Complete case analysis would not be problematic if it can be assumed that missingness occurs completely at random (34). This is, however, a strong assumption. When some data are available for those subsequently lost due to attrition, multiple imputation or inverse probability weighting can be used to reduce, or even remove, the possible selection bias. Some other alternative approaches have also been discussed (34–37).

We hypothesised that differences in hazards between participants and those lost due to attrition would change with time. Our study, however, did not support this hypothesis, which suggests that relative changes of unmeasured risk-factors in responders compared to withdrawers/non-responders were either absent, or not sufficiently large to influence outcomes.

Our study has limitations. Due to the way in which the data were collected up to wave 4, we were unable to distinguish withdrawal from non-response in analysis 1. If the magnitude of associations with mortality differed between withdrawal and non-response up to wave 4, our results in the analysis 2 might not generalise across all waves of the study. Because of the small number of deaths for each specific cause, we pooled all non-CVD deaths, which may have resulted in a diluted hazard since aetiology certainly differs across diseases. Cognitive impairment, a considerable determinant of the attrition (38), may have a major influence particularly in ageing cohort studies. However, we were unable to examine associations between cognitive function, attrition, and mortality because cognitive function was measured only from wave 5, by which time about three-quarters of the total attrition had already occurred. Although some results from the Whitehall II study could apply to more general populations (39), it would be interesting to repeat this work in a general population cohort to examine whether the association of response status with mortality is also reproducible. Further research on cause-specific mortality, such as subtypes of cancer, is required to estimate the hazard by response status in longitudinal studies.

In conclusion, these findings suggest that those who are lost due to attrition, no matter when attrition occurs, have an excess mortality within three to five years. Attrition, therefore, does have the potential to cause bias in follow-up studies. The response rate could be an indicator of selection bias, however not always (4, 33). We therefore recommend that researchers report characteristics of those excluded from the study to allow readers to evaluate the validity of findings, and consider applying statistical methodologies to minimise bias due to attrition.

## Supplementary Material

Supplementary File

## Figures and Tables

**Table 1.** Response status and cumulative death (CVD, all-cause) at each wave

Wave	Period	Participants (responders)	Attrition^a^			Cumulative CVD death (%) ^d^	Cumulative all-cause death
			*Cumulative Withdrawal (%)*^b^	*Non-response (%)*^b^	*Total (%)*^c^		
1	1985-1988	**10 308**	-	-	-	-	-
2	1989-1990	**8132**	2127 (20.7)^e^		**2127 (20.7)**	14 (28.6)	**49**
3	1991-1994	**8815**	1368 (13.4)^e^		**1368 (13.4)**	36 (28.8)	**125**
4	1995-1996	**8628**	774 (52.4)	712 (47.6)	**1486 (14.7)**	59 (30.4)	**194**
5	1997-1999	**7870**	882 (41.3)	1250 (58.7)	**2132 (21.3)**	95 (31.0)	**306**
6	2001	**7355**	975 (38.7)	1553 (61.3)	**2528 (25.6)**	132 (31.1)	**425**
7	2002-2004	**6967**	1246 (45.2)	1511 (54.8)	**2757 (28.4)**	176 (30.1)	**584**
8	2006	**7173**	1310 (55.5)	1051 (44.5)	**2361 (24.8)**	226 (29.2)	**774**
9	2007-2009	**6761**	1354 (52.2)	1239 (47.8)	**2593 (27.7)**	271 (28.4)	**954**
11^f^	2012-2013	**6308**	1389 (53.7)	1197 (46.3)	**2586 (29.1)**	405 (28.6)	**1414**
12	2015-2016	**5632**	1433 (49.7)	1448 (50.3)	**2881 (33.8)**	485 (27.0)	**1795**
*Deaths to August 2017*						*519 (26.7)*	***1943***

a Deaths are displayed separately from attrition (non-response or withdrawal)

b % of each attrition = [withdrawal or non-response / total attrition at each wave] * 100

c % attrition = [total attrition at each wave / (10308 - cumulative deaths at each wave)] * 100

d % CVD death = (CVD death / all-cause death) * 100

e Only pooled attrition is available at waves 2 and 3

f Wave 10 was a small pilot study of measures to be included at wave 11, and has not been included here

**Table 2.** Characteristics of study population (n=10 012)

	*n (%)*	Response rate (95%CI)^a^
**Sex**		
Men	6749 (67.4)	81.9 (81.7-82.2)
Women	3263 (32.6)	74.0 (73.6-74.5)
**Age in years**		
39 and below	2750 (27.5)	79.9 (79.5-80.4)
40 - 44	2607 (26.0)	80.0 (79.6-80.5)
45 - 49	2031 (20.3)	78.8 (78.2-79.3)
50 and over	2624 (26.2)	78.5 (78.0-79.0)
**Ethnicity**		
White	8968 (89.6)	80.9 (80.7-81.2)
Non-white	1044 (10.4)	65.8 (64.9-66.7)
**Marital status**		
Married/cohabit	7435 (74.3)	80.7 (80.4-81.0)
Single	1640 (16.4)	76.3 (75.7-77.0)
Divorced/widowed	937 (9.4)	74.0 (73.1-74.9)
**Employment grade**		
High	2979 (29.8)	86.1 (85.7-86.4)
Intermediate	4837 (48.3)	81.1 (80.7-81.4)
Low	2196 (21.9)	66.2 (65.5-66.8)
**Smoking habit**		
Never-smoker	4966 (49.6)	80.7 (80.4-81.1)
Ex-smoker	3225 (32.2)	81.3 (80.9-81.7)
Current smoker	1821 (18.2)	71.8 (71.1-72.4)
**Alcohol drinking**		
<14 units per week	7338 (73.3)	78.4 (78.2-78.7)
≥14 units per week	2674 (26.7)	81.9 (81.5-82.4)
**Physical activity**		
High	2175 (21.7)	80.9 (80.4-81.4)
Intermediate	2620 (26.2)	80.9 (80.5-81.4)
Low	5217 (52.1)	77.9 (77.6-78.3)

a Response rate = [number of waves responded / number of waves that it was possible to attend while still alive]*100

**Table 3.** Sub-distribution hazard ratios (SHRs) of CVD and Non-CVD mortality from wave 1 to August 2017, by attrition status^a^ (n=10 012)

			SHR (95% CI)			
			*Adjusted for*			
Outcome	Attrition status	No. deaths	*Sex and Age*		*All factors*^b^	
**CVD mortality**		*495*				
	Response	312		*ref.*		*ref.*
	Withdrawal/Non-response	183	1.86	(1.53-2.24)	1.55	(1.26-1.89)
**Non-CVD mortality**		*1367*				
	Response	873		*ref.*		*ref.*
	Withdrawal/Non-response	494	1.62	(1.45-1.82)	1.56	(1.39-1.76)

a Attrition status is time dependent and varies at each wave of the study

b Adjusted for sex, age, ethnicity, marital status, employment grade, smoking habit, alcohol drinking, and physical activity

## Summary Box

### What is already unknown on this topic

- Non-participation at baseline is known to be associated with increased risk of all-cause mortality.
- However, it is uncertain whether this finding is generalisable to attrition during follow-up in multi-wave longitudinal studies.
- Also, it is unknown whether attrition predicts cardiovascular mortality, and whether the association differs between two forms of attrition; non-response and withdrawal.

### What this study adds

- Participants lost due to attrition, no matter when attrition occurs in the study, have approximately 1.5 times higher mortality within three to five years than responders. Attrition, therefore, does have the potential to cause bias in follow-up studies.
- We recommend that researchers report characteristics of those excluded from the study to allow readers to evaluate the validity of findings, and consider applying statistical methodologies in analyses to minimise selection bias due to attrition.

## References

[1] Rothman KJ, Gallacher JE, Hatch EE. Why representativeness should be avoided. International journal of epidemiology. 2013;42(4):1012–4. 24062287 10.1093/ije/dys223 PMC3888189

[2] Rothman KJ, Greenland S, Lash TL. Modern epidemiology: Lippincott Williams & Wilkins; 2008.

[3] Porta M A dictionary of epidemiology: Oxford university press; 2014.

[4] Munafo MR, Tilling K, Taylor AE, Evans DM, Davey Smith G. Collider scope: when selection bias can substantially influence observed associations. Int J Epidemiol. 2018;47(1):226–35. 29040562 10.1093/ije/dyx206 PMC5837306

[5] Greenland S Quantifying Biases in Causal Models: Classical Confounding vs Collider-Stratification Bias. Epidemiology (Cambridge, Mass). 2003;14(3):300–6. 12859030

[6] Galea S, Tracy M. Participation rates in epidemiologic studies. Annals of epidemiology. 2007;17(9):643–53. 17553702 10.1016/j.annepidem.2007.03.013

[7] Morton LM, Cahill J, Hartge P. Reporting participation in epidemiologic studies: a survey of practice. Am J Epidemiol. 2006;163(3):197–203. 16339049 10.1093/aje/kwj036

[8] Mein G, Johal S, Grant RL, Seale C, Ashcroft R, Tinker A. Predictors of two forms of attrition in a longitudinal health study involving ageing participants: an analysis based on the Whitehall II study. BMC medical research methodology. 2012;12:164. 23106792 10.1186/1471-2288-12-164 PMC3505167

[9] Fekete C, Segerer W, Gemperli A, Brinkhof MW, Swi SCISG. Participation rates, response bias and response behaviours in the community survey of the Swiss Spinal Cord Injury Cohort Study (SwiSCI). BMC medical research methodology. 2015;15:80. 26450702 10.1186/s12874-015-0076-0 PMC4599658

[10] Matthews FE, Chatfield M, Freeman C, McCracken C, Brayne C. Attrition and bias in the MRC cognitive function and ageing study: an epidemiological investigation. BMC public health. 2004;4:12. 15113437 10.1186/1471-2458-4-12 PMC419705

[11] Hayward RD, Krause N. Forms of Attrition in a Longitudinal Study of Religion and Health in Older Adults and Implications for Sample Bias. Journal of Religion and Health. 2016;55(1):50–66. 25257794 10.1007/s10943-014-9949-5 PMC4375067

[12] Drivsholm T, Eplov LF, Davidsen M, Jorgensen T, Ibsen H, Hollnagel H, Representativeness in population-based studies: a detailed description of non-response in a Danish cohort study. Scandinavian journal of public health. 2006;34(6):623–31. 17132596 10.1080/14034940600607616

[13] Chatfield MD, Brayne CE, Matthews FE. A systematic literature review of attrition between waves in longitudinal studies in the elderly shows a consistent pattern of dropout between differing studies. Journal of clinical epidemiology. 2005;58(1):13–9. 15649666 10.1016/j.jclinepi.2004.05.006

[14] Goldberg M, Chastang JF, Zins M, Niedhammer I, Leclerc A. Health problems were the strongest predictors of attrition during follow-up of the GAZEL cohort. Journal of clinical epidemiology. 2006;59(11):1213–21. 17027433 10.1016/j.jclinepi.2006.02.020

[15] Harald K, Salomaa V, Jousilahti P, Koskinen S, Vartiainen E. Non-participation and mortality in different socioeconomic groups: the FINRISK population surveys in 1972-92. Journal of epidemiology and community health. 2007;61(5):449–54. 17435214 10.1136/jech.2006.049908 PMC2465683

[16] Jousilahti P, Salomaa V, Kuulasmaa K, Niemela M, Vartiainen E. Total and cause specific mortality among participants and non-participants of population based health surveys: a comprehensive follow up of 54 372 Finnish men and women. Journal of epidemiology and community health. 2005;59(4):310–5. 15767385 10.1136/jech.2004.024349 PMC1733044

[17] Stringhini S, Sabia S, Shipley M, Brunner E, Nabi H, Kivimaki M, Association of socioeconomic position with health behaviors and mortality. Jama. 2010;303(12):1159–66. 20332401 10.1001/jama.2010.297 PMC2918905

[18] Vega S, Benito-Leon J, Bermejo-Pareja F, Medrano MJ, Vega-Valderrama LM, Rodriguez C, Several factors influenced attrition in a population-based elderly cohort: neurological disorders in Central Spain Study. Journal of clinical epidemiology. 2010;63(2):215–22. 19473811 10.1016/j.jclinepi.2009.03.005

[19] Damen NL, Versteeg H, Serruys PW, van Geuns RJ, van Domburg RT, Pedersen SS, Cardiac patients who completed a longitudinal psychosocial study had a different clinical and psychosocial baseline profile than patients who dropped out prematurely. European journal of preventive cardiology. 2015;22(2):196–9. 24065741 10.1177/2047487313506548

[20] Leening MJ, Heeringa J, Deckers JW, Franco OH, Hofman A, Witteman JC, Healthy volunteer effect and cardiovascular risk. Epidemiology (Cambridge, Mass). 2014;25(3):470–1. 10.1097/EDE.0000000000000091 24713887

[21] Ferrie JE, Kivimaki M, Singh-Manoux A, Shortt A, Martikainen P, Head J, Non-response to baseline, non-response to follow-up and mortality in the Whitehall II cohort. International Journal of Epidemiology. 2009;38(3):831–7. 19264846 10.1093/ije/dyp153 PMC2722814

[22] Hara M, Sasaki S, Sobue T, Yamamoto S, Tsugane S. Comparison of cause-specific mortality between respondents and nonrespondents in a population-based prospective study: ten-year follow-up of JPHC Study Cohort I. Japan Public Health Center. Journal of clinical epidemiology. 2002;55(2):150–6. 11809353 10.1016/s0895-4356(01)00431-0

[23] Candido E, Kurdyak P, Alter DA. Item nonresponse to psychosocial questionnaires was associated with higher mortality after acute myocardial infarction. Journal of clinical epidemiology. 2011;64(2):213–22. 20566265 10.1016/j.jclinepi.2010.03.010

[24] Delgado-Rodríguez M, Llorca J. Bias. Journal of epidemiology and community health. 2004;58(8):635–41. 15252064 10.1136/jech.2003.008466 PMC1732856

[25] Walker M, Shaper A, Cook D. Non-participation and mortality in a prospective study of cardiovascular disease. Journal of epidemiology and community health. 1987;41(4):295–9. 3455423 10.1136/jech.41.4.295 PMC1052650

[26] Kivimäki M, Batty GD, Pentti J, Shipley MJ, Sipilä PN, Nyberg ST, Association between socioeconomic status and the development of mental and physical health conditions in adulthood: a multi-cohort study. The Lancet Public Health. 2020;5(3):e140–e9. 32007134 10.1016/S2468-2667(19)30248-8

[27] Health Do. Alcohol Guidelines Review–Report from the Guidelines Development Group to the UK Chief Medical Officers. Department of Health London; 2016.

[28] Wærsted M, Børnick TS, Twisk JWR, Veiersted KB. Simple descriptive missing data indicators in longitudinal studies with attrition, intermittent missing data and a high number of follow-ups. BMC Research Notes. 2018;11(1):123. 29433533 10.1186/s13104-018-3228-6 PMC5809924

[29] Van Loon AJ, Tijhuis M, Picavet HS, Surtees PG, Ormel J. Survey non-response in the Netherlands: effects on prevalence estimates and associations. Annals of epidemiology. 2003;13(2):105–10. 12559669 10.1016/s1047-2797(02)00257-0

[30] Corry NH, Williams CS, Battaglia M, McMaster HS, Stander VA. Assessing and adjusting for non-response in the Millennium Cohort Family Study. BMC medical research methodology. 2017;17(1):16. 28129735 10.1186/s12874-017-0294-8 PMC5273843

[31] Fernandez-Ballesteros R, Zamarron MD, Diez-Nicolas J, Lopez-Bravo MD, Molina MA, Schettini R. Mortality and refusal in the longitudinal 90+ project. Archives of gerontology and geriatrics. 2011;53(2):e203–8. 20943279 10.1016/j.archger.2010.09.007

[32] Eaton WW, Anthony JC, Tepper S, Dryman A. Psychopathology and attrition in the epidemiologic catchment area surveys. Am J Epidemiol. 1992;135(9):1051–9. 1595691 10.1093/oxfordjournals.aje.a116399

[33] Gustavson K, Røysamb E, Borren I. Preventing bias from selective non-response in population-based survey studies: findings from a Monte Carlo simulation study. BMC medical research methodology. 2019;19(1):120. 31195998 10.1186/s12874-019-0757-1 PMC6567536

[34] Daniel RM, Kenward MG, Cousens SN, De Stavola BL. Using causal diagrams to guide analysis in missing data problems. Stat Methods Med Res. 2012;21(3):243–56. 21389091 10.1177/0962280210394469

[35] Welch CA, Sabia S, Brunner E, Kivimäki M, Shipley MJ. Does pattern mixture modelling reduce bias due to informative attrition compared to fitting a mixed effects model to the available cases or data imputed using multiple imputation?: a simulation study. BMC medical research methodology. 2018;18(1):89. 30157752 10.1186/s12874-018-0548-0 PMC6114233

[36] Greenland S, Pearl J, Robins JM. Causal Diagrams for Epidemiologic Research. Epidemiology (Cambridge, Mass). 1999;10(1):37–48. 9888278

[37] Daniel RM, De Stavola BL, Cousens SN, Vansteelandt S. Causal mediation analysis with multiple mediators. Biometrics. 2015;71(1):1–14. 25351114 10.1111/biom.12248 PMC4402024

[38] Van Beijsterveldt CE, van Boxtel MP, Bosma H, Houx PJ, Buntinx F, Jolles J. Predictors of attrition in a longitudinal cognitive aging study: the Maastricht Aging Study (MAAS). Journal of clinical epidemiology. 2002;55(3):216–23. 11864790 10.1016/s0895-4356(01)00473-5

[39] Batty GD, Shipley M, Tabak A, Singh-Manoux A, Brunner E, Britton A, Generalizability of occupational cohort study findings. Epidemiology (Cambridge, Mass). 2014;25(6):932–3. 10.1097/EDE.0000000000000184 25265141

