# Supplementary material for: Association of attrition with mortality: Findings from 11 waves over three decades of the Whitehall II study
Source: J Epidemiol Community Health. Author manuscript; Available in PMC 2021 Oct 1. (PMC8071845; doi:10.1136/jech-2019-213175)
Supplement: Supplementary File [file NIHMS1692543-supplement-Supplementary_File.pdf]

Table S1. Association of attrition status at each wave with CVD and Non-CVD mortality up to the subsequent wave. (Analysis 1)

			CVD mortality		Non-CVD mortality	
**Wave**	**Response status**	**No. alive**	**No. deaths**	**SHR (95% CI)[a]**	**No. deaths**	**SHR (95% CI)[a]**
**1**	**Responders**	10 012	12	-	29	-
**2**	**Responders**	8024	12	*ref.*	35	*ref.*
	**Withdrawal/Non-response**	1947	9	3.39 (1.35-8.53)	18	2.18 (1.24-3.84)
**3**	**Responders**	8647	20	*ref.*	38	*ref.*
	**Withdrawal/Non-response**	1250	3	1.30 (0.38-4.40)	5	0.89 (0.35-2.26)
**4**	**Responders**	8462	25	*ref.*	57	*ref.*
	**Withdrawal/Non-response**	1369	9	2.38 (1.11-5.11)	15	1.53 (0.85-2.73)
**5**	**Responders**	7723	23	*ref.*	51	*ref.*
	**Withdrawal/Non-response**	2002	9	1.65 (0.75-3.63)	24	1.86 (1.14-3.04)
**6**	**Responders**	7231	28	*ref.*	69	*ref.*
	**Withdrawal/Non-response**	2387	15	1.59 (0.84-3.01)	41	1.77 (1.21-2.61)
**7**	**Responders**	6855	27	*ref.*	77	*ref.*
	**Withdrawal/Non-response**	2610	22	2.29 (1.29-4.07)	62	2.00 (1.43-2.80)
**8**	**Responders**	7054	28	*ref.*	92	*ref.*
	**Withdrawal/Non-response**	2223	16	1.74 (0.95-3.18)	38	1.27 (0.87-1.85)
**9**	**Responders**	6655	73	*ref.*	183	*ref.*
	**Withdrawal/Non-response**	2448	55	1.99 (1.40-2.84)	133	1.88 (1.51-2.34)
**11**	**Responders**	6213	43	*ref.*	178	*ref.*
	**Withdrawal/Non-response**	2446	36	1.84 (1.15-2.93)	112	1.47 (1.17-1.85)
**12[b]**	**Responders**	5551	21	*ref.*	64	*ref.*
	**Withdrawal/Non-response**	2739	9	0.84 (0.38-1.86)	46	1.27 (0.86-1.86)
P-value for linearity				*P=0.11*		*P=0.61*

[a] Adjusted for sex and age

[b] Mortality follow-up from wave 12 is up to August 2017

Table S2. Sub-distribution hazard ratios (SHRs) of CVD and Non-CVD mortality from wave 1 to August 2017, by attrition status[a] in 10 012 participants (*person years as time-scale*)

Outcome	Attrition status	No. deaths	SHR (95% CI) Adjusted for *Sex and Age*		*All factors*[b]	
**CVD mortality**		*495*				
	Response	312		*ref.*		*ref.*
	Withdrawal/Non-response	183	1.76	(1.45-2.13)	1.46	(1.20-1.79)
**Non-CVD mortality**		*1367*				
	Response	873		*ref.*		*ref.*
	Withdrawal/Non-response	494	1.54	(1.38-1.73)	1.48	(1.32-1.67)

[a] Attrition status is time dependent and varies at each wave of the study

[b] Adjusted for sex, age, ethnicity, marital status, employment grade, smoking habit, alcohol drinking, and physical activity

Table S3. Sub-distribution hazard ratios (SHRs) and 95% confidence interval (CIs) of CVD and non-CVD mortality by response status from wave 4 to August 2017 in 8791 participants (Analysis 2)

Outcome	Response status	No. Deaths	SHR (95% CI) Adjusted for *Sex and Age*		*p-value*[a]	*+Demography and health risk behaviours*[b]		*p-value*[a]	*+General health status*[c]		*p-value*[a]
**CVD mortality**		*353*									
	Response	258	*ref.*			*ref.*			*ref.*		
	Withdrawal	33	1.28	(0.89-1.84)	*0.102*	1.14	(0.79-1.65)	*0.218*	1.21	(0.84-1.75)	*0.284*
	Non-response	62	1.82	(1.37-2.41)		1.49	(1.10-2.01)		1.53	(1.13-2.06)	
**Non-CVD mortality**		*1056*									
	Response	748	*ref.*			*ref.*			*ref.*		
	Withdrawal	136	1.75	(1.46-2.11)	*0.617*	1.72	(1.43-2.08)	*0.593*	1.77	(1.47-2.13)	*0.377*
	Non-response	172	1.65	(1.40-1.95)		1.62	(1.36-1.92)		1.59	(1.34-1.89)	

[a] P-value of Likelihood Ratio Test between the model with attrition status (response and withdrawal/non-response) and response status (response, withdrawal, non-response)
[b] Additionally adjusted for ethnicity, marital status, employment grade, smoking habit, alcohol drinking, and physical activity
[c] Additionally adjusted for PCS and MCS from each wave

Table S4. SHRs and 95% CIs of CVD mortality in three models (Analysis 2)

		Adjusted for				
	Sex and age		+ Demography and health behaviours		+ Health status	
n=8791	SHR	95% CI	SHR	95% CI	SHR	95% CI
Response status						
Response		*ref.*		*ref.*		*ref.*
Withdrawal	1.28	(0.89-1.84)	1.14	(0.79-1.65)	1.21	(0.84-1.75)
Non-response	1.82	(1.37-2.41)	1.49	(1.10-2.01)	1.53	(1.13-2.06)
Sex						
Men		*ref.*		*ref.*		*ref.*
Women	0.78	(0.62-0.98)	0.57	(0.43-0.75)	0.54	(0.40-0.71)
Age in years						
39 and below		*ref.*		*ref.*		*ref.*
40 - 44	1.58	(1.01-2.47)	1.59	(1.01-2.49)	1.50	(0.95-2.36)
45 - 49	3.29	(2.17-5.00)	3.20	(2.09-4.88)	2.79	(1.83-4.25)
50 and over	7.33	(5.03-10.66)	7.20	(4.92-10.54)	6.02	(4.12-8.81)
Ethnicity						
White				*ref.*		*ref.*
Non-white			1.49	(1.08-2.05)	1.42	(1.02-1.96)
Marital status						
Married/cohabit				*ref.*		*ref.*
Single			1.46	(1.10-1.92)	1.47	(1.12-1.95)
Divorced/widowed			0.96	(0.67-1.39)	0.95	(0.66-1.37)
Employment grade						
High				*ref.*		*ref.*
Intermediate			1.07	(0.82-1.40)	1.05	(0.81-1.38)
Low			1.50	(1.05-2.14)	1.47	(1.03-2.11)
Smoking habit						
Never-smoker				*ref.*		*ref.*
Ex-smoker			1.11	(0.87-1.42)	1.10	(0.86-1.41)
Current smoker			1.62	(1.23-2.14)	1.54	(1.17-2.03)
Alcohol drinking						
<14 units per week				*ref.*		*ref.*
≥14 units per week			0.90	(0.69-1.16)	0.90	(0.70-1.16)
Physical activity						
High				*ref.*		*ref.*
Intermediate			0.97	(0.69-1.36)	0.95	(0.68-1.34)
Low			1.34	(1.00-1.78)	1.31	(0.98-1.74)
SF-36: PCS						
Q4 (best)						*ref.*
Q3					1.61	(1.01-2.35)
Q2					1.42	(0.97-2.09)
Q1 (worst)					2.39	(1.68-3.40)
SF-36: MCS						
Q4 (best)						*ref.*
Q3					0.84	(0.63-1.11)
Q2					0.72	(0.53-0.96)
Q1 (worst)					0.74	(0.56-0.98)

Table S5. SHRs and 95% CIs of non-CVD mortality in three models (Analysis 2)

	Sex and age		*Adjusted for* + Demography and health behaviours		+ Health status	
n=8791	SHR	95% CI	SHR	95% CI	SHR	95% CI
Response status						
Response		*ref.*		*ref.*		*ref.*
Withdrawal	1.75	(1.46-2.11)	1.72	(1.43-2.08)	1.77	(1.47-2.13)
Non-response	1.65	(1.40-1.95)	1.62	(1.36-1.92)	1.59	(1.34-1.89)
Sex						
Men		*ref.*		*ref.*		*ref.*
Women	0.94	(0.83-1.07)	0.95	(0.81-1.11)	0.90	(0.76-1.05)
Age in years						
39 and below		*ref.*		*ref.*		*ref.*
40 - 44	1.30	(1.04-1.63)	1.30	(1.04-1.64)	1.29	(1.03-1.63)
45 - 49	2.25	(1.82-2.79)	2.33	(1.88-2.89)	2.22	(1.78-2.77)
50 and over	4.56	(3.78-5.50)	4.76	(3.93-5.77)	4.45	(3.65-5.42)
Ethnicity						
White				*ref.*		*ref.*
Non-white			0.75	(0.60-0.94)	0.69	(0.55-0.87)
Marital status						
Married/cohabit				*ref.*		*ref.*
Single			1.00	(0.84-1.20)	0.97	(0.82-1.16)
Divorced/widowed			1.05	(0.86-1.28)	1.02	(0.84-1.24)
Employment grade						
High				*ref.*		*ref.*
Intermediate			1.02	(0.88-1.17)	0.99	(0.86-1.15)
Low			0.89	(0.73-1.09)	0.83	(0.68-1.02)
Smoking habit						
Never-smoker				*ref.*		*ref.*
Ex-smoker			1.09	(0.94-1.25)	1.06	(0.92-1.22)
Current smoker			2.04	(1.75-2.37)	1.91	(1.64-2.23)
Alcohol drinking						
<14 units per week				*ref.*		*ref.*
≥14 units per week			1.10	(0.96-1.26)	1.10	(0.96-1.26)
Physical activity						
High				*ref.*		*ref.*
Intermediate			0.81	(0.68-0.97)	0.80	(0.67-0.96)
Low			0.96	(0.83-1.12)	0.92	(0.79-1.07)
SF-36: PCS						
Q4 (best)						*ref.*
Q3					1.20	(0.97-1.49)
Q2					1.42	(1.16-1.74)
Q1 (worst)					2.04	(1.69-2.46)
SF-36: MCS						
Q4 (best)						*ref.*
Q3					1.01	(0.85-1.20)
Q2					1.18	(0.99-1.39)
Q1 (worst)					1.32	(1.12-1.56)
